# Interactions between glioblastoma and myeloid cells

**DOI:** 10.3389/fcell.2025.1632122

**Published:** 2025-06-24

**Authors:** Yuting Li, Yuhong Chen, Kai Cai, Yujuan Qin, Xi Wang, Bo Zhang, Lin Shi, Zonglin He, Jiasheng Wang, Jiecun Long, Yishun Zeng, Qiong Gong

**Affiliations:** ^1^ Guangxi University of Chinese Medicine, Nanning, China; ^2^ The First Affiliated Hospital Of GuangxiUniversity Of Chinese Medicine, Nanning, China; ^3^ Department of Nephrology, The Second Affiliated Hospital of Guangxi Medical University, Nanning, Guangxi, China; ^4^ Institute of Cardiovascular Sciences, The People’s Hospital of Guangxi Zhuang Autonomous Region & Guangxi Academy of Medical Sciences, Nanning, China; ^5^ School of Medicine, Guangxi University, Nanning, China

**Keywords:** GBM, TAM, MDSC, TME, myeloid cells

## Abstract

Standing as the most aggressive form of primary malignant tumor, Glioblastoma (GBM) tumors with marked heterogeneity represents one of the enormous challenges in glioma treatment. Myeloid cells, which includes neutrophils, myeloid-derived suppressor cells, microglia, and macrophages, play a pivotal role in the tumor microenvironment of GBM. In the tumor microenvironment (TME), T cells and natural killer (NK) cells exert anti-tumor functions, whereas myeloid-derived suppressor cells (MDSCs) can promote tumor progression by suppressing these immune responses. Therefore, MDSCs play a critical role in shaping the effectiveness of immunotherapy. TME has constrained the ability of traditional GBM treatment approaches to significantly enhance prognostic outcomes for patients. This category encompasses conventional therapies like surgical resection and radiation therapy, along with cutting-edge methodologies such as immunotherapy. Through extensive investigations into the dynamic interactions between the GBM microenvironment and neoplastic cells, both targeted treatment strategies and innovative immunotherapeutic modalities have emerged, offering promising new directions for clinical intervention. This review focuses on the interactions between GBM and myeloid cells (MCs), providing novel insights into the oncogenesis and progression of GBM.

## 1 Introduction

Glioblastoma is regarded as the most aggressive brain tumor (Roda and Bottone, 2022). For GBM, treatment includes radiotherapy, chemotherapy, and targeted therapies. Despite these approaches, the prognosis for GBM patients remains grim, with one contributing factor being the presence of an immunosuppressive TME (Qazi et al., 2017). Studies have identified interactions between glioblastoma and the TME, which play a role in mediating immune suppression within GBM (de Groot et al., 2020; Doucette et al., 2013; Klemm et al., 2020; Pang et al., 2022; Sanmamed and Chen, 2018; Sharma and Allison, 2015; Wang et al., 2017).

Myeloid cells are a key component of the immune landscape within the GBM tumor microenvironment and constitute an integral part of the tumor tissue. Initially, MC infiltrating GBM were termed glioma-associated macrophages/small glial cells (GAMs) or tumour-associated macrophages (TAMs), and their heterogeneity was not fully understood at that time (Gieryng et al., 2017; Quail and Joyce, 2017; Wei et al., 2013). With the increasing identification of specific markers for myeloid cell subsets, the heterogeneity of MCs in GBM and their role in immune suppression are being gradually uncovered (Table 1). Based on differences in intrinsic gene expression, GBM can be classified into three subtypes: classical, proneural, and mesenchymal (Verhaak et al., 2010; Wang et al., 2019), which exhibit significant differences in molecular characteristics, clinical behaviour, and potential therapeutic targets. Each GBM subtype displays distinct levels of heterogeneity within the TME. Beyond cell-intrinsic mechanisms, cancer cell signalling also influences and interacts with components of the TME. In turn, the tumour microenvironment can facilitate GBM progression and contribute to resistance against both chemotherapy and immunotherapy (White et al., 2023). The remarkable efficacy of immunotherapy has been demonstrated across multiple cancer types, highlighting its significant therapeutic potential. However, its effectiveness in treating GBM has been disappointing, which is closely linked to the immunosuppressive nature of the TME. Studies have revealed that the interactive relationship between glioma cells and the TME contributes to shaping immune heterogeneity within the microenvironment (Klemm et al., 2020; Wu P. et al., 2023). Inter-tumoral and intra-tumoral heterogeneity provides evidence for unraveling GBM progression and treatment resistance.

**TABLE 1:** Overview of myeloid cells.

Cell type	Origin	Main function or feature
Microglia	CNS-resident	Pro-/anti-inflammtory roles; metabolic redulation
Macrophages	Bone marrow-derived	M1 (pro-inflammatory), M2 (pro-tumor)polarization
M-MDS	Monocytic origin	Express CD74; immunosuppressive
PMN-MDSCs	Neutophil-like	Recruited via CXCL1/2-dependent mechanisms
E-MDSCs	Early undifferentiated precursors	Active glycolysis and lipid metabolism
Neutrophils	Skull bone marrow	Express MHC class II; potential anti-tumor function
Dendritic Cells	Hematopoietic Stem Cells	Involved in immune regulation
Eosinophils	Multipotent hematopoietic stem cells	Pro-inlfammatory, chemotactic, cytotoxic
Basophils	Multipotent hematopoietic stem cells	Secreting inflammatory mediators, regulating Th2 immunity

The most abundant immune cell populations in TME components mainly include TAMs and microglia (Chen et al., 2017). TAMs not only facilitates the development and progression of GBM but also contributes to its treatment resistance. Meanwhile, signal transduction in cancer cells and the TME exhibit bidirectional interactions (Zhao et al., 2019; Xuan et al., 2021). However, therapies solely targeting TAMs have not significantly improved patient prognosis.

This review provides an overview of the roles myeloid cells play within the GBM microenvironment, how GBM influences these cells, and the reciprocal effects myeloid cells exert on tumor progression. Furthermore, we discuss emerging single-cell approaches that enable the identification of novel TAM subpopulations potentially critical to GBM development and immune evasion.

### 1.1 The role of myeloid cells in the GBM microenvironment

**FIGURE 1:**
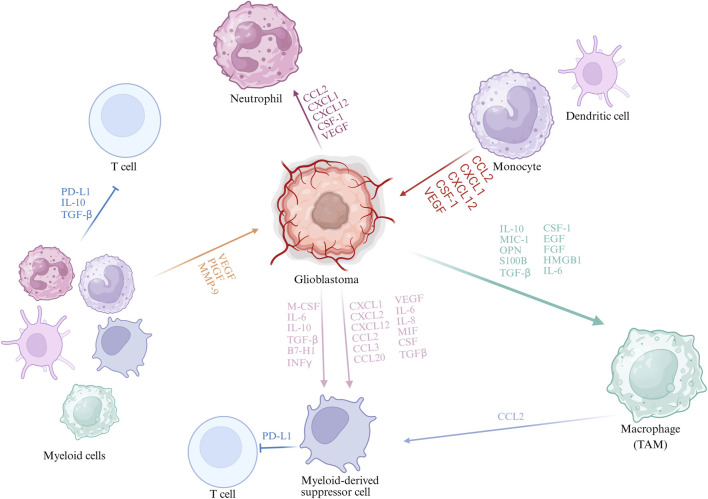
Interactions Between GBM and Myeloid Cells. During GBM progression, tumour cells secrete cytokines and chemokines (e.g., CCL2, CXCL12, CSF-1, VEGF) that recruit myeloid cells including monocytes, neutrophils, and MDSCs. Distinct TAM subpopulations with specific gene signatures have been identified, such as microglia-like, hypoxic, and phagocytic macrophages. TAM-derived CCL2 recruits CCR2^+^Ly6C^+^ monocytic MDSCs, enhanced by tumour factors like osteoprotegerin, promoting immunosuppression. Myeloid cells suppress anti-tumour immunity via cytokines (TGF-β, IL-10) and chemokines (CCL2, CXCL12), facilitating GBM immune evasion.

TAMs, predominantly originating from peripheral blood mononuclear cells, are the most abundant myeloid cells within the GBM microenvironment and are generally categorized into pro-inflammatory M1 and anti-inflammatory M2 (Xuan et al., 2021). M2 TAMs dominate in GBM, promoting tumour growth, angiogenesis, and immune suppression (Quail and Joyce, 2017; Joseph et al., 2022; Sarkar et al., 2014). The M2 can be further subdivided into M2a, M2b, and M2c. M2a macrophages, induced by IL-4 and IL-13, are involved in tissue repair, angiogenesis, and extracellular matrix remodeling. M2b macrophages, activated by immune complexes and Toll-like receptor (TLR) signaling, exhibit partial pro-inflammatory activity but also secrete IL-10 to maintain an immunosuppressive environment. M2c macrophages, mainly induced by IL-10 and glucocorticoids, contribute to immune tolerance and tissue remodeling, and display enhanced immunosuppressive and pro-metastatic functions in tumors (Li et al., 2022; Vidyarthi et al., 2019). Figure 1 illustrates the interactions between glioblastoma and myeloid cells.

TAMs in brain tumors exhibit diversity and heterogeneity. Single-cell analysis studies have shown that TAMs differ in composition between primary and metastatic brain tumors (Blanco-Carmona et al., 2023). For example, CD64^+^, CD11c^+^, CX3CR1^+^, Mertk^+^, and CD49d^−^ are markers of reactive microglia (Ye et al., 2023). These microglia are widely distributed in the tumor regions of primary brain tumors such as GBM, but not in the core areas of metastatic brain tumors. Conversely, in both GBM and brain metastases, macrophages tend to localize around CD31^+^ vascular structures (!!! INVALID CITATIONb). Moreover, single-cell analyses have reinforced the concept of TAM heterogeneity in GBM, revealing multiple subpopulations with distinct genetic signatures in both human patients and mouse models. These include transient state macrophages (characterized by high expression of LYZ, EREG, S100A6, and low C1Q), glia-like macrophages (expressing BIN1, CX3CR1, TMEM119, OLFML3), hypoxic macrophages (marked by BNIP3, ADAM8, FAM162A, MIF), and phagocytic/lipid-rich macrophages (with elevated levels of FABP5, GPNMB, LGALS3, CD63), among others. Growing evidence suggests that TAM heterogeneity in GBM is highly dependent on the microenvironmental context (Pombo Antunes et al., 2021). Differences between primary and recurrent tumors are a major contributing factor to the heterogeneity of TAMs. Studies have shown that in newly diagnosed GBM, microglia constitute the predominant myeloid population, whereas in recurrent GBM, macrophages become the dominant component (Abdelfattah et al., 2022).

## 2 Regulation of myeloid cells by GBM

GBM exhibits a highly intricate TME that is heavily populated by various myeloid-derived immune cells (Kesarwani et al., 2019). These myeloid subsets are integral to driving GBM progression, enabling immune evasion, and contributing to treatment resistance (refer to Table 2). Through a range of regulatory pathways, GBM influences the recruitment, phenotypic polarization, and immunosuppressive functionality of these cells, thereby constructing a TME that favors tumor persistence and expansion (Table 3).

**TABLE 2:** Pro-tumor effects of myeloid cells on GBM.

Function	Example of mechanisms
Tumor Growth	Creatine synthesis; secretion of VEGF/EGF/IL-6; activation of GLUT1 and STAT3 pathways
Immune Suppression	Expression of PD-L1; secretion of IL-10/TGF- β; induction of T cell exhaustion via BLIMP-1
Angiogenesis	VEGF, IL-8, mmp9, Hif α-LGMN axis, IL-1
Regulatiom of GSCs	VEGF activates endothelia cells → increased adhesion molecules → enhanced myeloid cell infiltration

**TABLE 3:** GBM-mediated regulation of myeloid cells.

Regulation type	Example of Molecules/Mechanisms
Chemokines	CCL2/CCR2, CXCL1/2/8, CSF-1, CXCL12
Cytokines	IL-10, TGF-β(induce immunosuppressive polarization)
Exosomal Factors	miR-21, miR-29a, PD-L1, TGF-β (induced immunosuppression polarization)
Vascular Mechanisms	VEGF activates endothelia cells → increased adhesion molecules
Epigenetic Regulation	→ enhanced myeloid cell infiltration Demethylation of Irf8 in GSCs uregulates Ccl9,promoting myeloid cell infiltration

### 2.1 Cytokines and chemokines

GBM cells secrete a spectrum of cytokines and chemokines—including CCL2, CXCL1, CXCL12, colony-stimulating factor 1 (CSF-1), and vascular endothelial growth factor (VEGF)—which interact with corresponding receptors on various myeloid cell types, thereby promoting their recruitment into the TME (Magod et al., 2021; Veglia et al., 2021; Zha et al., 2020; Atai et al., 2011; Bronte et al., 2016; Gao et al., 2021). Among these, CCL2 is particularly crucial for directing monocytes from peripheral blood into the tumor, where engagement with CCR2 initiates their differentiation into TAMs (Magod et al., 2021; Guo et al., 2019; Qiu et al., 2021). Immune-driven epigenetics can facilitate the recruitment of TAMs and enhance the immune heterogeneity of the TME, primarily by activating pathways involved in myeloid cell recruitment.

In the context of tumor development, Myeloid-Derived Suppressor Cells (MDSCs) play a fundamental role by being actively recruited in response to chemokine gradients established by tumor-secreted factors. The CXCR2–CXCL5 signaling axis plays a prominent role in mediating MDSC infiltration, particularly by directing the accumulation of monocytic MDSCs (M-MDSCs) (Zha et al., 2020; Atai et al., 2011). Additionally, the infiltration of polymorphonuclear MDSCs (PMN-MDSCs) is driven by cues such as CCL2, CCL3, and hypoxic stress. IL-8 functions as a potent enhancer of MDSC mobilization from the bone marrow (Lad et al., 2024). In brain metastasis settings, CXCL10 plays a key role in shaping the pre-metastatic niche and contributes to immune evasion mechanisms within the brain tumor environment.

TAMs can regulate the tumor microenvironment and impair T cell function by releasing immunosuppressive mediators, including IL-10, as well as various chemokines (Pang et al., 2023). Meanwhile, hypoxia-inducible factor 1-alpha (HIF-1α) can induce the expression of the protease legumain (LGMN) in TAMs, thereby enhancing their immunosuppressive capacity. Neutrophils can also exert anti-tumor effects in GBM, primarily by stimulating T cell responses through MHC II molecules (Sarantopoulos et al., 2024). In GBM, both TAMs and MDSCs contribute to immune evasion by secreting immunomodulatory factors such as TGF-β, CCL2, and CXCL12, which promote the recruitment and polarization of regulatory immune cells. In addition, glioblastoma stem cells (GSCs) enhance the infiltration of immunosuppressive myeloid cells such as macrophages and MDSCs by upregulating Ccl9 expression through epigenetic modifications, including Irf8 gene demethylation, thereby contributing to the establishment of an immune-privileged microenvironment. Furthermore, IFNγ signaling pathway may promote tumor cell activation through cytokine-mediated mechanisms, leading to immune escape (Lad et al., 2024). The development of nanomedicine platforms—such as those delivering the cytokine LIGHT or modulating cannabidiol (CBD)—has also shown potential in enhancing intratumoral chemokine expression, increasing effector T cell infiltration, and alleviating myeloid cell-mediated immunosuppression.

### 2.2 Extracellular vesicles (EVs)

While infiltrating the tumor microenvironment in GBM, MDSCs are also activated by immunoregulatory cytokines such as macrophage colony-stimulating factor (M-CSF) and granulocyte-macrophage colony-stimulating factor (GM-CSF) (He et al., 2025). During the progression of GBM, not only soluble factors play a role, but GBM-derived extracellular vesicles also exert significant influence, including promoting tumor development and enhancing the activation of MDSCs. Under hypoxic stress, exosomes secreted by GBM cells facilitate MDSC proliferation by transporting specific microRNAs—including miR-1246, miR-29a, and miR-92a. These exosomal miRNAs are internalized by local MDSCs, where they initiate downstream signaling through the DUSP3/ERK axis, leading to functional activation (Qiu et al., 2021). GBM-infiltrating MDSCs also release PD-L1-enriched exosomes, which are taken up by B cells via caveolae-dependent endocytosis, thereby enhancing PD-L1 expression in B cells (Shurin and Umansky, 2022), which further weakening host antitumor immunity and supporting GBM progression. Moreover, MDSCs act as essential mediators in the cross-talk between tumor cells and T lymphocytes. For instance, GBM-derived EVs have been shown to selectively drive M-MDSC expansion, ultimately impairing T cell-mediated immune surveillance. Beyond signaling and transcriptional regulation, the immunosuppressive function of MDSCs is also maintained by their altered metabolism in GBM (Wang et al., 2025); Specifically, the production of polyamines and lipids sustains their suppressive capacity. Targeting the arginine–ornithine–polyamine metabolic circuit impairs MDSC viability and function, thereby enhancing anti-tumor immunity and inhibiting tumor progression. These findings contribute to a better understanding of the role of MDSCs in immune suppression and tumor progression during GBM development.

Exosomes serve as essential vehicles for intercellular communication, delivering functional biomolecules—including proteins and various RNAs—that modulate the physiological state of target cells. In the context of GBM, vesicles secreted by tumor cells can be internalized by myeloid-derived populations, thereby transmitting tumor-promoting cues. Importantly, GBM-derived exosomes are capable of carrying immunoregulatory cytokines, which facilitate the reprogramming of myeloid cells into immunosuppressive phenotypes, consequently enhancing immune evasion and facilitating tumor progression (Hong et al., 2021). In addition, exosomal cargo may include metabolic regulators such as non-coding RNAs and enzymes that influence key metabolic pathways—including those related to hypoxia adaptation and glycolytic activity—within recipient myeloid cells, thereby supporting tumor proliferation (Pyonteck et al., 2013). Moreover, these extracellular vesicles can also participate in chemotactic signaling, actively recruiting MDSCs or TAMs into the tumor niche by transporting chemokines such as CCL2 and CXCL12, further contributing to the establishment of a protumoral microenvironment.

Current research reveals that GBM modulates myeloid cells through exosome-mediated mechanisms encompassing signal transduction, metabolic control, and targeted therapeutic approaches. GBM promotes immunosuppression in myeloid populations by transferring pro-tumorigenic factors like IL-1β and TGF-β via exosomes. The molecular cargo of these vesicles, including microRNAs and metabolic enzymes, contributes to the reprogramming of myeloid cell metabolism (Kesarwani et al., 2019). Furthermore, bioengineered exosomes offer precise modulation of myeloid cell activities, presenting potential to reverse the immunosuppressive environment characteristic of GBM.

Moreover, exosomes serve as critical modulators of tumour-associated myeloid cell activity, including microglia and macrophages, thereby facilitating the development of an immunosuppressive tumour microenvironment (Liu et al., 2025). GBM cells actively secrete exosomes enriched with diverse bioactive contents—such as microRNAs (miRNAs), proteins, and metabolic intermediates—that significantly influence the recruitment, differentiation, and polarisation of myeloid populations. For example, GBM-derived exosomes containing miR-21 and miR-29a have been implicated in enhancing monocyte migration toward tumour regions. In addition, the immunosuppressive cargo of these vesicles, notably molecules like TGF-β and PD-L1, contributes to the attenuation of antitumour immune responses (Zhao et al., 2022).

Genetically engineered microglial exosomes have been designed to display immune checkpoint antibodies, such as anti-LAG3, on their surfaces while simultaneously being loaded with photosensitizing agents (Youssef et al., 2025). These modified exosomes possess the ability to traverse the blood–brain barrier and selectively home to the GBM tumor microenvironment, where they modulate immune functions. Their crossing of the blood–brain barrier is mediated by integrin proteins VLA-4 and LFA-1, facilitating targeted delivery of therapeutics to specific sites. Additionally, the presence of anti-LAG3 on the exosome surface counteracts T cell exhaustion, thereby reinstating antitumor immune activity. When used in conjunction with photothermal therapy, these engineered exosomes markedly improve treatment outcomes against GBM (Mehdizadeh et al., 2025).

### 2.3 Vascular endothelial cells

**FIGURE 2:**
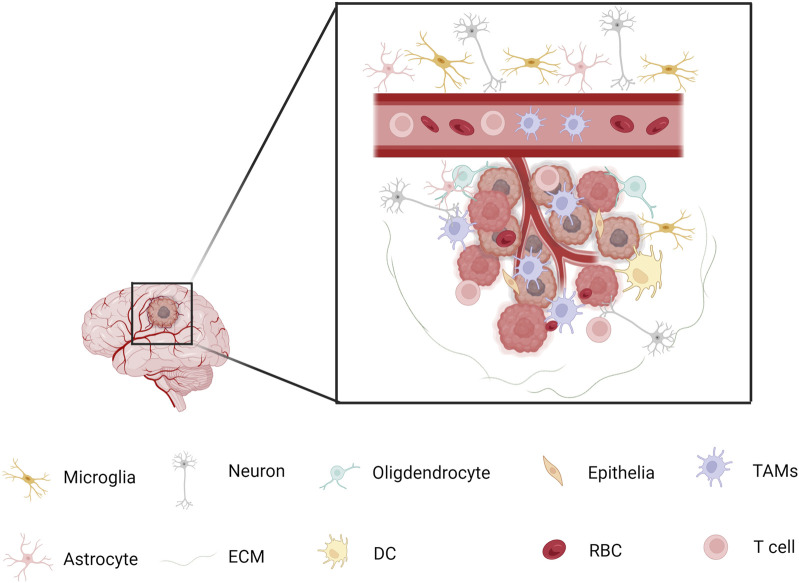
Spatial Relationships Among Different Cell Types in GBM Microglia and macrophage populations tend to exhibit spatial segregation. Macrophages are more likely to cluster with each other and remain distant from microglia, while microglia are enriched near other microglia and avoid macrophages. However, some myeloid cell subpopulations—such as TAM-Cd68, TAM-Int, and Mg-like cells—are distributed more randomly and evenly throughout the tumor microenvironment, showing minimal distance differences relative to other cell types. Macrophages tend to cluster in regions with high tumor cell density and phenotypic diversity, where they interact with various non-myeloid cell types. Almost all myeloid populations show a significantly higher proportion of interactions with core tumor cells, as well as increased interactions with glial cells located at the tumor margin.

GBM cells promote the extravasation and infiltration of myeloid cells through interactions with vascular endothelial cells. Specifically, VEGF secreted by GBM not only drives angiogenesis but also increases the expression of adhesion molecules, including ICAM-1 and VCAM-1, on endothelial cell surfaces. This heightened expression facilitates stronger binding of myeloid cells to the vascular endothelium, thereby aiding their migration into tumor tissues (Shaw et al., 2024). The spatial distribution and interactions among various cell populations in GBM are depicted in Figure 2.

Lymphatic endothelial cell (LEC)-like populations have been identified in GBM tissues. These cells secrete the chemokine CCL21, which interacts with the CCR7 receptor on GSCs, enhancing cholesterol metabolism and promoting GSC proliferation (Kidwell and Aghi, 2024). While most studies have focused on the regulatory mechanisms of GSCs, LECs, as a subpopulation of endothelial cells, may also indirectly influence the recruitment or function of myeloid cells within the tumor microenvironment through the secretion of chemokines. Such effects are potentially mediated by paracrine signaling and may contribute to the remodeling of the immunosuppressive microenvironment. Dysfunctional endothelial cells can release cytokines such as VEGF and CCL2, which not only support angiogenesis but also activate inflammatory signaling pathways that attract TAMs (Shen et al., 2021). Specific populations of immunosuppressive myeloid cells are enriched in the pseudopalisading regions of IDH-wildtype GBM, which are characterized by hypoxia and cellular stress. These cells colocalize with stem-like tumor cells and engage in metabolic and signaling interactions through axes such as CXCL8–CXCR1 and FGF11–FGFR1 (Jackson et al., 2025). Although the direct regulatory role of endothelial cells remains unclear, vascular abnormalities in pseudopalisading regions may indirectly influence the metabolic plasticity and immunosuppressive behavior of myeloid cells through mechanisms involving hypoxia-inducible factors (HIFs). Moreover, angiogenesis-related cytokines, including IL-8 and TGF-β, may contribute to the recruitment and activation of myeloid cells, although the precise mechanisms require further investigation (Bae et al., 2022). Figure 2 illustrates the spatial distribution and interactions of various cell types within the GBM microenvironment.

## 3 Effects of myeloid cells on GBM

### 3.1 Promoting tumour growth

Myeloid cells promote GBM progression through several pathways, including hypoxia-induced creatine synthesis and uptake by tumor cells. They also suppress T cell activity through immunosuppressive molecules such as IL-10, thereby promoting the formation of an immunosuppressive tumor microenvironment. Furthermore, monocytes and TAMs release pro-angiogenic factors such as VEGF, supporting neovascularization. In addition to these effects, myeloid cells secrete a range of cytokines and growth factors that can directly or indirectly stimulate GBM cell proliferation and tumor expansion.

Tumor vasculature plays a critical supportive and nutritive role during GBM development, with myeloid cell-derived VEGF contributing to its rapid expansion. In addition, EGF and IL-6 further promote tumor proliferation and enhance its survival capacity (Pudełek et al., 2020).

Studies have shown that tumour-associated myeloid cells (TAMCs) in hypoxic regions of GBM synthesise creatine via HIF1α-dependent metabolic pathways (Rashidi et al., 2024). Tumour cells absorb creatine via SLC6A8 to enhance energy metabolism, promoting proliferation and maintaining stemness. Targeting creatine uptake inhibitors (such as GPA) significantly inhibits tumour growth and prolongs survival (Gangoso et al., 2021). In addition, inhibition of CSF-1R effectively depletes TAMs and extends survival (Pyonteck et al., 2013). Monocyte-derived TAMs (Mo-TAMs) highly express hypoxia-related genes, promoting angiogenesis and inhibiting T cell proliferation; targeting CSF1R drugs (such as PLX3397) can reduce TAM infiltration and inhibit tumour growth (Priceman et al., 2010). In the pseudoproliferative zones of IDH-WT GBM, myeloid inhibitory cells (E-MDSC and M-MDSC) interact with tumour stem cells via the CXCL8-CXCR1 and FGF11-FGFR1 signalling pathways, promoting metabolic adaptation and immune suppression (Jackson et al., 2025; Sun et al., 2023).

### 3.2 Immune suppression

Myeloid cells facilitate tumor immune evasion by secreting a variety of factors as well as suppress anti-tumour immune responses through multiple mechanisms, thereby indirectly promoting GBM growth.

MDSCs play a crucial role in dampening T cell activity and weakening anti-tumor immune defenses. Research demonstrates that MDSCs release arginase and inducible nitric oxide synthase (iNOS), enzymes that deplete arginine and produce nitric oxide, effectively hindering T cell proliferation (Bian et al., 2018). Moreover, myeloid cells can drive macrophage polarization toward the M2 phenotype, with these M2 macrophages exhibiting immunosuppressive properties and facilitating tumor progression (Liu et al., 2021; Wu M. et al., 2023).

Studies have identified two immunosuppressive myeloid subpopulations within IDH wild-type GBM: early myeloid-derived suppressor cells (E-MDSCs) and M-MDSCs. E-MDSCs display metabolic features akin to cancer cells, including enhanced glycolysis and fatty acid metabolism, which enable their survival in hypoxic and nutrient-limited tumour microenvironments while suppressing T cell proliferation. These cells secrete FGF11 to activate the FGFR1 signalling pathway, thereby promoting the expansion of tumour stem-like cells and showing a strong correlation with T cell exhaustion. Within the hypoxic ‘pseudo-barrier’ zones of GBM, E-MDSCs co-localize with tumour stem-like cells, creating an immunosuppressive niche through chemokine-mediated interactions such as CXCL8-CXCR1. Additionally, hypoxia-induced protease LGMN is markedly overexpressed in TAMs, where it triggers the GSK-3β–STAT3 signalling cascade, driving TAMs to polarize towards an immunosuppressive phenotype and consequently impairing CD8^+^ T cell infiltration and activity (Raghavan et al., 2021). Inhibiting LGMN can reverse immune suppression and improve the therapeutic response to anti-PD-1 treatment. Moreover, TAMs located in hypoxic areas increase the expression of genes involved in glycolysis, such as GLUT1 and HK2, along with pathways related to fatty acid oxidation, which supports their survival and stimulates the release of immunosuppressive cytokines including IL-10 and TGF-β (Gharzeddine et al., 2024).

Myeloid cells drive GBM immune suppression through multiple mechanisms. They promote tumour stem cell proliferation and suppress T cell effector functions through pathways such as FGF11-FGFR1, thereby regulating MDSC metabolic adaptation and signal interactions. Additionally, myeloid cells can induce immunosuppressive phenotypes through LGMN and hypoxia signals, participating in TAM polarisation and protease regulation. By modulating IL-10 and the JAK/STAT pathway, they directly lead to T cell functional exhaustion. The sphingolipid signal of TREM2 reprograms central specificity to regulate TAM phenotypes (Jiang et al., 2025).

### 3.3 Angiogenesis

Myeloid cells contribute to angiogenesis within the GBM microenvironment through the secretion of angiogenic molecule which collectively facilitate the delivery of essential nutrients. The multidimensional mechanisms by which myeloid cells promote GBM angiogenesis include: direct secretion of proangiogenic factors (e.g., VEGF, IL-8); extracellular matrix (ECM) remodelling (via proteases such as MMP-9); hypoxia-driven signalling pathways (HIF-1α-LGMN axis); paracrine loops (IL-1β/IL-1R1); and indirect support for VM formation (via regulation of CSCs and EMT). Through the aforementioned mechanisms, myeloid cells directly or indirectly promote GBM growth and progression, making them important targets in GBM therapy.

Myeloid cells facilitate new vessel growth by producing MMP-9, an enzyme that breaks down ECM components, thereby enabling endothelial sprouting and migration independent of VEGF signaling. In glioblastoma, elevated MMP-9 levels strongly correlate with enhanced angiogenesis and tumor invasiveness. Research demonstrates that TAMs contribute to endothelial cell migration and neovascularization by degrading ECM collagen, such as type IV collagen, via MMP-9 secretion. Moreover, antisense RNA-mediated suppression of MMP-9 markedly decreases tumor vascular density in nude mouse models, highlighting MMP-9 as a promising therapeutic target (Khan et al., 2023). In the glioblastoma microenvironment, hypoxia triggers the increased expression of HIF-1α, which drives TAMs toward an immunosuppressive phenotype and elevates levels of the protease LGMN, thereby enhancing angiogenesis induced by low oxygen conditions. Additionally, HIF-1α directly regulates key angiogenic genes, including VEGFR1 and VEGF-A, further enhancing angiogenesis and increasing tumour cell invasiveness. Studies have found that the IL-1β/IL-1R1 paracrine circuit promotes the expression of angiogenic factors. Myeloid cells (such as BMDM) and GBM cells form a feedforward loop via the IL-1β/IL-1R1 signal. IL-1β activates the NF-κB pathway in tumour cells, inducing the secretion of monocyte chemotactic proteins (MCPs), which further recruit more myeloid cells, forming a vicious cycle of angiogenesis and immune suppression. In mouse models, antagonising IL-1β or IL-1R1 reduces TAM infiltration and inhibits tumour angiogenesis, significantly prolonging survival (Chen et al., 2023). Promoting the formation of vascular mimicking (VM). VM is a tumor-derived vascular-like structure that provides an independent blood supply pathway for GBM independent of traditional blood vessels. Myeloid cell-derived factors, including TGF-β and IL-10,which can indirectly facilitate VM by influencing CSC characteristics or EMT processes. Notably, VM exhibits inherent resistance to anti-angiogenic agents like bevacizumab, underscoring the necessity for combination therapies that specifically target VM-related pathways—such as blocking HIF-1α or EMT-associated signaling—to enhance treatment efficacy (Zhang et al., 2023).

## 4 Treatment strategies

### 4.1 Targeting TAMs

Targeting the CSF-1R or CCL2/CCR2 signaling pathways can effectively diminish the recruitment and activity of TAMs. TREM2, a critical receptor expressed on TAM surfaces, plays a distinctive protective role in GBM. Insights from single-cell and spatial transcriptomic analyses reveal a negative association between TREM2 expression and both immunosuppressive myeloid populations and T cell exhaustion within GBM. Sphingolipid-rich molecules in the central nervous system, such as sphingomyelin (SM) and glycosphingolipids (GSLs), can interact with the TREM2 receptor on TAMs, promoting a phenotypic shift from the pro-tumoral M2 type to the anti-tumoral M1 type, thereby significantly inhibiting the progression of GBM (!!! INVALID CITATIONe).

Nanoformulations reshape the immune-suppressive microenvironment mediated by TAMs. The ‘Nano-reshaper’ nanomedicine developed by Chen Jun’s team at Fudan University delivers CBD and the cytokine LIGHT to systemically enhance immune function and locally modulate the TME. CBD mitigates immune suppression by reducing TAM-derived secretion of IL-10 and TGF-β, whereas LIGHT facilitates vascular normalization and upregulates chemokines that attract lymphocytes, leading to increased infiltration of effector T cells. In mouse models, anti-PD-L1 therapy has demonstrated significant efficacy by attenuating TAM-mediated immunosuppression, highlighting its potential as a therapeutic strategy (Molgora et al., 2020).

Tumor-associated TAMswithin GBM promote mesenchymal transition and invasive behavior of GSCs by releasing the pro-invasive molecule TGFβI, also known as BIGH3. This protein is markedly upregulated in aggressive GBM subtypes and correlates with unfavorable patient prognosis. Suppression of BIGH3 signaling—through the use of antibodies or small-molecule inhibitors—effectively diminishes the invasive potential of GSCs (Yan et al., 2022). Antibody therapy targeting BIGH3 has shown promising results in reducing tumor invasion within 3D engineered tumor models and animal studies, indicating its potential as a viable approach to postpone GBM recurrence (Youssef et al., 2025).

Treatment strategies targeting TAMs in GBM must account for their remarkable heterogeneity and context-dependent plasticity. Current approaches primarily include: regulating key receptors (e.g., TREM2, LGMN) to reverse TAM polarisation; blocking pro-invasive factors (e.g., BIGH3) to inhibit tumour progression; using nanotechnology for drug delivery to remodelling systemic and local immunity; and combination therapies (e.g., ICB, metabolic inhibitors) to overcome drug resistance. However, clinical translation faces substantial challenges, including limited blood-brain barrier (BBB) permeability and the difficulty of selectively targeting specific TAM subpopulations, whose dynamic responses to microenvironmental shifts significantly contribute to therapeutic resistance. Future research could leverage single-cell multi-omics combined with spatial transcriptomics to thoroughly characterize the functional subsets of TAMs and unravel the regulatory networks governing their behavior.

### 4.2 Targeting MDSCs

GBM actively facilitate the infiltration of MDSCs by releasing various chemotactic factors, including CCL20, interleukin-8 (IL-8) (Sarantopoulos et al., 2024; Lad et al., 2024; He et al., 2025). Moreover, CXCL1 and CXCL2 play a promotive role in the recruitment of M-MDSCs and polymorphonuclear cells (Shurin and Umansky, 2022; Wang et al., 2025; Hong et al., 2021). While MDSCs and TAMs represent distinct populations within the GBM microenvironment, emerging evidence suggests they are functionally interconnected and may participate in a continuum of immunosuppressive differentiation. TAMs upon stimulation by soluble mediators such as osteoprotegerin and CCL20 secreted by GBM cells, produce CCL2, which in turn recruits CCR2^+^ Ly6C^+^ M-MDSCs into the TME, exacerbating local immune suppression (Hong et al., 2021). Several studies have demonstrated that M-MDSCs, under the influence of tumor-derived cytokines such as IL-6 and GM-CSF, can differentiate into M2-like TAMs, further amplifying immunosuppression and promoting tumor progression (Takacs et al., 2024; Bayik et al., 2020). This phenotypic transition highlights a dynamic interplay between these cell types and underscores the need for therapeutic strategies that concurrently target both MDSC-mediated suppression and TAM reprogramming.

MDSCs exhibit marked heterogeneity and play multifaceted roles in the progression of tumors. These cells contribute to tumor angiogenesis, enhance invasiveness, and facilitate immune evasion, primarily by inhibiting the activity of T cells and natural killer (NK) cells (Dapash et al., 2021). Targeting the early MDSCs and TAMs interaction network. IDH wild-type GBM contains a distinct population of E-MDSCs with metabolic profiles resembling those of cancer cells is one of the therapeutic approaches. These E-MDSCs interact with stem cell-like tumour cells via the FGF11–FGFR1 signalling pathway, fostering an immunosuppressive microenvironment. E-MDSCs frequently co-localise with TAMs and recruit additional myeloid inhibitory cells through chemokines such as IL-6. Therapeutically targeting the metabolic pathways of E-MDSCs—such as glycolysis or fatty acid oxidation—or blocking key chemokine axes like CXCL8–CXCR1 may suppress their pro-tumour activities and restore T cell-mediated antitumour immunity. Furthermore, disrupting the metabolic pathways that support the activity of MDSCs represents a promising approach to impair their immunosuppressive capabilities. Targeting these essential metabolic routes including glycolysis and fatty acid can effectively undermine MDSC-mediated immune inhibition. In IDH wild-type GBM, E-MDSCs promote tumour stem cell proliferation and inhibit T cell activity through the FGF11-FGFR1 signalling pathway. Targeting key glycolytic enzymes like lactate dehydrogenase A (LDHA) or using fatty acid oxidation inhibitors such as etomidate can impair MDSC function. Preclinical studies suggest that combining these metabolic inhibitors with immune checkpoint blockade (ICB) therapies significantly enhances antitumour efficacy (Miska and Chandel, 2023).

The receptor CD300ld, predominantly plays a crucial role in modulating their recruitment and immunosuppressive activity via the STAT3–S100A8/A9 signaling pathway, which is mainly expressed on polymorphonuclear myeloid-derived suppressor cells (PMN-MDSCs). In mouse models, inhibiting CD300ld—either via administration of its extracellular domain protein or through gene knockout—markedly suppresses tumour progression, including glioblastoma. Humanised mouse studies further validate the conservation and therapeutic potential of CD300ld as an immunotherapy target.

Inhibiting the exosome-driven miR-21 signaling pathway represents a promising therapeutic strategy. Studies have shown that blocking miR-21 can disrupt the exosome-mediated positive feedback loop orchestrated by glioblastoma-associated mesenchymal stem cells (GA-MSCs). Interruption of this loop attenuates MDSC infiltration and weakens the immunosuppressive nature of the tumor microenvironment.

Facilitating the maturation of MDSCs into fully differentiated immune cells offers a strategy to counteract their immunosuppressive effects, which are largely attributable to their immature phenotype. Research has shown that agents such as all-trans retinoic acid (ATRA) and vitamin D3 can effectively promote the differentiation of MDSCs into functional macrophages or dendritic cells, thereby significantly reducing their ability to suppress immune responses. Additionally, combining ATRA with chemotherapeutic agents like temozolomide has demonstrated improved therapeutic outcomes in GBM models. Clinical investigations further support that ATRA administration decreases MDSC levels in patients and enhances overall immune function (Tobin et al., 2023).

At present, several immune evasion-related pathways and targets have been identified as therapeutic entry points in GBM,wich includes. For instance, blockade of TREM2, CSF1R, or PI3Kγ can reprogram immunosuppressive TAMs, while targeting CXCR1/2 or IDO1 may inhibit MDSC recruitment and function (Sun et al., 2023; Zhong et al., 2024; Li et al., 2020). Metabolic checkpoints such as Arginase-1 and STAT3 are also critical regulators of immune suppression, and their inhibition has shown potential to synergize with immune checkpoint blockade therapies.

The way to counteract immune evasion also involves targeting immunosuppressive molecules produced by MDSCs, which can impair T cell activity, including transforming growth factor-beta (TGF-β),arginase 1 (Arg-1) and inducible nitric oxide synthase (iNOS). Blocking the expression or activity of these factors can effectively restore T cell function and restore T cell killing capacity. Small molecule inhibitors (e.g., CB-1158 targeting Arg-1) or neutralising antibodies (e.g., anti-TGF-β antibodies) have demonstrated efficacy in GBM models and have entered early clinical trial phases.

GBM treatment strategies targeting MDSCs must account for their heterogeneity and dynamic regulatory characteristics, with key directions including: specific receptor blockade (e.g., CD300ld, CCR2) to inhibit MDSC recruitment; signal pathway intervention (e.g., STAT3, miR-21) to reverse immune suppression; metabolic reprogramming to weaken MDSC survival capacity; and combination therapies (e.g., immunotherapy, chemotherapy) to overcome drug resistance. Clinical application of MDSC-targeted therapies faces several key obstacles, including limited permeability across the blood–brain barrier, the need for precise targeting of distinct MDSC subtypes, and the ability of these cells to dynamically adapt to evolving microenvironmental cues that drive therapeutic resistance. To overcome these challenges, future research should incorporate single-cell multi-omics alongside spatial transcriptomics to provide a more comprehensive understanding of the functional heterogeneity within MDSC populations and uncover the molecular pathways governing their regulatory roles.

### 4.3 Immune checkpoint inhibitors

Within the TME, myeloid cells can drive immunosuppression through the secretion of cytokines such as IL-10 and TGF-β. Under hypoxic stress, TAMs upregulate LGMN expression via HIF-1α signalling, subsequently activating the GSK-3β–STAT3 cascade, thereby further dampening T cell functionality. Pharmacological targeting of LGMN counteracts this suppressive effect and, when combined with anti-PD-1 therapy, markedly extends survival in GBM preclinical models. Additionally, E-MDSCs found in IDH wild-type GBM facilitate the expansion of glioma stem-like cells through the FGF11–FGFR1 signalling pathway and release inhibitory mediators such as Arg-1, which depletes essential metabolites from the T cell milieu, impairing their cytotoxic activity and undermining the efficacy of ICIs.

To improve the therapeutic response of immune checkpoint inhibitors (ICIs) in GBM, a growing body of research supports the combination of ICIs with treatments specifically aimed at counteracting the immunosuppressive roles of myeloid cells. One promising approach involves disrupting the HIF1α–LGMN signalling axis in TAMs, which reduces the accumulation of immunosuppressive TAMs in the tumour microenvironment and boosts CD8^+^ T cell-mediated antitumour activity. Experimental models have demonstrated that LGMN inhibition, when paired with anti-PD-1 antibodies, leads to a marked reduction in tumour progression. Another innovative strategy employs a nanomedicine platform known as ‘Nano-reshaper’, which co-delivers CBD and the cytokine LIGHT. This formulation suppresses IL-10 production by TAMs, supports vascular normalisation, and enhances effector T cell infiltration, thereby potentiating the efficacy of anti-PD-1 treatment. In parallel, targeting the metabolic pathways sustaining myeloid-derived suppressor cell (MDSC) function—such as inhibiting glycolysis through LDHA blockers or suppressing fatty acid oxidation with agents like etomidate—has shown promise. Preclinical findings suggest that combining such metabolic interventions with ICIs results in significantly improved antitumour responses (Hossain et al., 2015).

Immune checkpoint inhibitors have achieved therapeutic success in various cancers, but their efficacy in glioblastoma has been relatively limited. To overcome this obstacle, advances in nanotechnology—such as the development of functionalized liposomes—have been explored to facilitate the trans-BBB transport of ICIs, thereby potentially improving their accessibility and therapeutic impact within the GBM microenvironment. For example, transferrin receptor antibody-modified nanoparticles can target and deliver anti-PD-1 antibodies to GBM lesions (Ramalho et al., 2022). Optimising treatment timing and sequence: TMZ chemotherapy may offset the efficacy of ICIs by reducing tumour-infiltrating lymphocytes (TILs). Studies indicate that adjusting the administration sequence of TMZ and ICIs (e.g., ICIs followed by chemotherapy) can preserve immune memory and enhance treatment efficacy (Sampson et al., 2020). Dynamic immune suppression network: Myeloid cells in GBM exhibit high plasticity, and ICIs may induce their phenotypic conversion (e.g., TAMs from M1 to M2 polarisation). Combining TAMs polarisation-regulating drugs (e.g., TREM2 agonists) may improve treatment resistance.

Novel biomarkers and personalised therapy. the application of PD-L1 in GBM faces significant challenges due to pronounced intratumoral heterogeneity. Notably, its expression is intricately influenced by the immunoregulatory functions of myeloid cells. For instance, TAMs can enhance PD-L1 levels on glioma cells through IL-10 secretion, thereby establishing an immunosuppressive barrier that facilitates immune evasion (Pu and Ji, 2022). Integrative analysis using single-cell sequencing alongside spatial transcriptomics has identified a subset of myeloid cells characterized by elevated expression of TREM2. Notably, the presence of this specific population shows a positive association with patient survival outcomes, highlighting its potential utility as a predictive biomarker for guiding combination immunotherapeutic strategies.

Myeloid cells act as major barriers to immune checkpoint inhibitor therapy in GBM through metabolic reprogramming, secretion of inhibitory factors, and dynamic phenotypic conversion. Future directions include: targeting myeloid cell-specific pathways (e.g., LGMN, TREM2); optimising combination therapy regimens (e.g., ICIs + metabolic inhibitors or nanodrugs); and developing novel biomarkers (e.g., single-cell multi-omics feature profiles) to guide personalised treatment. Table 4 provides the shows the classification of the therapeutic strategies.

**TABLE 4:** Classification of therapeutic strategies.

Strategy Catgory	Approach	Examples of Targets/Methods
Targeting TAMs	Polarization modulation Nanomedicine-based regulation Blocking pro-invasive signaling	TREM2 (promote M1), LGMN(promotes M2-liike polarization) CBD + LIGHT (Nano-reshaper) Anti-BIGH3 antibody to inhibit GSC mesenchymal transition and invasion
Targeting MDSCs	Inhibit recruitment	CCR2/CCL2 axis, CXCL8-CXCR1, CD300Id (PMN-MDSCs)
	Inhibit function Induce differentiation	miR-21 loop, STAT3, Arg-1, TGF- β, LDHA and other metabolic factors ATRA, vitamin D3 to promote differentiation into DCs/macrophages
ICIs Combination Therapy	Combined with myeloid modulators Combine with metabolic targeting Enhanced BBB pentration Optimized dosing schedule Suppor for personalized therapy	TREM2 agonist, LGMN inhibitor LDHA inhibition, blocking fatty acid oxidation Transferrin receptor-targeted Nanoparticles Administer ICIs prior to TMZ Based on biomarkers such as TREM2 expression, PD-L1 distribution and functional status

## 5 Summary and outlook

GBM represents a profoundly heterogeneous, aggressively invasive, and highly malignant tumor of the central nervous system. Although treatment approaches have progressively advanced, patient outcomes continue to be poor, highlighting the urgent need for a more comprehensive understanding of the disease’s pathophysiological basis and the creation of improved targeted therapeutic interventions. In the complex microenvironment of GBM, myeloid cells (primarily including bone marrow-derived myeloid immune cells such as macrophages, myeloid-derived inhibitory cells, dendritic cells, and neutrophils) constitute key participants in tumour-associated immune responses and play multifaceted roles in tumour initiation and progression, immune escape, and treatment resistance.

This review discusses the dynamic interactions between GBM and myeloid cells within the tumor microenvironment. Initially, it examines how GBM cells influence myeloid cells to adopt an immunosuppressive phenotype by secreting cytokines (including CSF-1, IL-10), releasing metabolic byproducts (including lactate and adenosine), and producing extracellular vesicles. These factors collectively foster a ‘tumor-supportive myeloid cell population’ that facilitates tumor progression. Subsequently, the review addresses the feedback role of myeloid cells in GBM, highlighting their critical contributions to angiogenesis, tumor migration and invasion, and the formation of immune evasion barriers. Furthermore, the discussion includes the utilization of cutting-edge methodologies like single-cell sequencing, integrated multi-omics analyses, and spatial transcriptomics to deepen the understanding of GBM-myeloid cell interactions.

Despite abundant research underscoring the role of myeloid cells in GBM, significant challenges remain that must be addressed before these findings can be successfully applied in clinical settings. First, the high heterogeneity and plasticity of myeloid cells make them difficult to regulate precisely with a single target. For example, the dynamic transition between different macrophage states poses a significant challenge to precise regulation. Second, the blood-brain barrier structure and highly immunosuppressive environment of GBM limit the penetration and efficacy of immunotherapy drugs (such as ICIs) in the brain. Third, most studies have focused on *in vitro* or animal models, and systematic research on clinical samples, especially human patient *in situ* samples, remains scarce.

Future research should focus on the following directions:

1. Deeply characterise the myeloid cell subpopulation landscape: Utilise single-cell multi-omics, spatial omics, and mass cytometry technologies to systematically characterise the distribution, dynamic changes, and functional states of various myeloid cells in the GBM microenvironment, establishing a high-resolution cell atlas to provide a precise foundation for targeted therapy.
2. Elucidating key regulatory pathways and interaction networks: Further explore the key signaling axes between GBM and myeloid cells and their upstream regulatory mechanisms, identify key regulatory nodes, and develop novel targets for intervention pathways, such as CSF-1R, CCL2-CCR2, TREM2, and PD-L1/PD-1.
3. Design more effective combined immunotherapy strategies: Considering that the immunosuppressive role of myeloid cells is one of the key reasons for the low efficacy of current GBM immunotherapy, future efforts may explore the combination of myeloid cell modulators with existing treatment modalities (such as ICIs, CAR-T, and radiotherapy) to enhance immune responses.
4. Develop intervention methods with higher delivery efficiency: Utilising nanotechnology and bioengineering materials, design drug delivery systems targeting myeloid cells to enhance blood-brain barrier penetration, improve treatment specificity, and ensure safety.
5. Emphasise clinical sample research and translational medicine: Strengthen collaboration with clinical practice to obtain real immune profiling information from patient surgical or biopsy tissues, and incorporate myeloid immune intervention strategies into clinical trials to facilitate the translation of basic discoveries into clinical applications.

**FIGURE 3:**
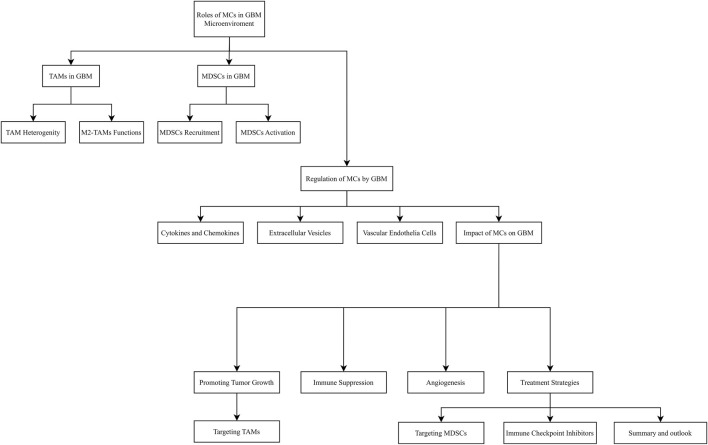
The relevant cellular interactions.

In summary, the bidirectional regulatory role of myeloid cells in the GBM microenvironment has become a research hotspot and a potential breakthrough area. As research into the complex interactions within the tumor microenvironment continues to deepen, new therapeutic opportunities and challenges are emerging, targeting myeloid cells holds promise as a key strategy to improve GBM treatment outcomes and enhance immunotherapy efficacy. However, this process requires multidisciplinary collaboration and parallel advancement of basic and clinical research to ultimately deliver substantial survival benefits for GBM patients in the future. Figure 3 outlines the relevant cellular interactions.
